# Metabolism-related proteins as biomarkers for predicting prognosis in polycystic ovary syndrome

**DOI:** 10.1186/s12953-024-00238-9

**Published:** 2024-12-19

**Authors:** Nan Ding, Ruifang Wang, Peili Wang, Fang Wang

**Affiliations:** The addresses of the institutions: Reproductive Medicine Center, Lanzhou University Second Hospital, No.82, Cuiying Road, Chengguan District, Lanzhou City, Gansu Province China

**Keywords:** Polycystic ovary syndrome, Quantitative proteomics, Metabolism-related proteins, Prognostic, Prediction model

## Abstract

**Objective:**

The study aimed to explore the role of metabolism-related proteins and their correlation with clinical data in predicting the prognosis of polycystic ovary syndrome (PCOS).

**Methods:**

This research involves a secondary analysis of proteomic data derived from endometrial samples collected from our study group, which includes 33 PCOS patients and 7 control subjects. A comprehensive identification and analysis of 4425 proteins were conducted to screened differentially expressed proteins (DEPs). Gene Ontology (GO) and Kyoto Encyclopedia of Genes and Genomes (KEGG) enrichment analyses were subsequently performed on the DEPs. To identify independent prognostic metabolism-related proteins, univariate Cox regression and LASSO regression were applied. The expression levels of these proteins were then used to develop a prognostic model, with their predictive accuracy evaluated through receiver operating characteristic (ROC) curves, decision curve analysis (DCA), and calibration curves. Furthermore, we also investigate the correlation between clinical data and prognostic proteins.

**Results:**

The study identified 285 DEPs between the PCOS and control groups. GO enrichment analysis revealed significant involvement in metabolic processes, while KEGG pathway analysis highlighted pathways such as glycolysis/gluconeogenesis and glucagon signaling. Ten key metabolism-related proteins (ACSL5, ANPEP, CYB5R3, ENOPH1, GLS, GLUD1, LDHB, PLCD1, PYCR2, and PYCR3) were identified as significant predictors of PCOS prognosis. Patients were separated into high and low-risk groups according to the risk score. The ROC curves for predicting outcomes at 6, 28, and 37 weeks demonstrated excellent predictive performance, with AUC values of 0.98, 1.0, and 1.0, respectively. The nomogram constructed from these proteins provided a reliable tool for predicting pregnancy outcomes. DCA indicated a net benefit of the model across various risk thresholds, and the calibration curve confirmed the model’s accuracy. Additionally, we also found BMI exhibited a significant negative correlation with the expression of GLS (*r* =-0.44, *p* = 0.01) and CHO showed a significant positive correlation with the expression of LDHB (*r* = 0.35, *p* = 0.04).

**Conclusion:**

The identified metabolism-related proteins provide valuable insights into the prognosis of PCOS. The protein based prognostic model offers a robust and reliable tool for risk stratification and personalized management of PCOS patients.

**Supplementary Information:**

The online version contains supplementary material available at 10.1186/s12953-024-00238-9.

## Introduction

Polycystic ovary syndrome (PCOS) is a common endocrine disorder that impacts about 5–10% of women in their reproductive years [1]. It is characterized by a spectrum of symptoms including oligo- or anovulation, hyperandrogenism, and polycystic ovarian morphology [2]. In addition to its well-documented reproductive implications, PCOS is closely linked to a range of metabolic abnormalities such as insulin resistance, obesity, and dyslipidemia [3, 4]. These metabolic disturbances exacerbate the overall disease burden and contribute to the complex pathophysiology of PCOS.

The crucial factors for reproductive success are the embryo, the endometrium, and the crosstalk between them [5, 6]. Current research on PCOS primarily aims at enhancing ovulatory function and managing symptoms related to hyperandrogenism [7, 8]. These approaches address some of the clinical fertility issues of PCOS, however, the underlying mechanisms leading to adverse reproductive outcomes remain less thoroughly explored. A growing body of evidence indicates that endometrial dysfunction may increase the risk of pregnancy complications in PCOS [9, 10]. Issues with decidualization can lead to implantation failure, miscarriage, pre-eclampsia, and premature delivery [11]. While metabolic dysfunctions, particularly those related to lipid and glucose metabolism, play a critical role in the pathogenesis and prognosis of PCOS. These metabolic disturbances impair endometrial receptivity, thereby influencing fertility outcomes and contributing to complications such as pregnancy loss and subfertility [11–13]. Therefore, understanding the molecular alterations in the endometrium associated with metabolic dysfunctions in PCOS is crucial for developing targeted interventions.

Quantitative proteomics has emerged as a valuable method for uncovering the intricate molecular mechanisms involved in various diseases, including PCOS [14–16]. By analyzing the proteomic profiles of endometrial tissues, it is possible to identify differentially expressed proteins (DEPs) that may serve as biomarkers for disease prognosis and therapeutic targets. This study investigated the significance of metabolism-related proteins in the prognosis of PCOS by conducting a comprehensive proteomic analysis of endometrial samples from PCOS patients and control subjects. We integrated the proteomic data with clinical outcomes to identify key proteins associated with adverse reproductive outcomes and constructed a predictive model. Our research enhances the understanding of metabolic dysregulation in PCOS and provides a basis for developing personalized treatment strategies to improve reproductive outcomes for affected women.

## Materials and methods

### Sample collection and protein samples preparation

This study involves a secondary analysis of proteomic data from endometrial samples collected at the Reproductive Center of the Second Hospital of Lanzhou University between September 2019 and September 2020. The cohort consisted of 33 patients diagnosed with polycystic ovary syndrome (PCOS) and 7 control women with successful pregnancies. PCOS diagnoses were based on the Rotterdam criteria, requiring at least two of the following: (1) oligo- or anovulation, (2) clinical and/or biochemical signs of hyperandrogenism, and (3) polycystic ovaries. Exclusion criteria included other endocrine disorders (e.g., hypothyroidism, hyperprolactinemia, adrenal disease), hypertension, diabetes, and recent use of medications affecting hormone or glucose metabolism. Controls were non-PCOS women with regular menstrual cycles and normal ovarian morphology as confirmed by ultrasound. Informed consent was obtained from all participants, and the study received approval from the Ethics Committee of Lanzhou University Second Hospital (No: 2017A-057). The endometrial samples were collected using the Pipelle endometrial aspirator (CooperSurgical, USA). Residual blood was removed by rinsing the tissue with phosphate-buffered saline (PBS). The cleaned samples were then divided: one part was placed into a cryopreservation tube and stored at -80℃ for future use, while the other part was sent for pathological examination. Histological analysis was conducted to confirm that the samples were taken during the proliferative phase. Frozen endometrial samples were homogenized using a tissue homogenizer to ensure complete disruption of the tissue. The homogenized samples were then lysed with sodium dodecyl sulfate (SDS) and dithiothreitol (DTT) buffer, sonicated to further break down cellular structures, and subsequently digested with trypsin. The peptides were desalted on C18 cartridges and subsequently analyzed using nano LC-MS/MS in both data-dependent and data-independent acquisition modes, with details provided below.

### Mass spectrometry assay for data dependent acquisition (DDA) and data independent acquisition (DIA)

Mass spectrometry was conducted using a Thermo Scientific Q Exactive HF X mass spectrometer coupled with an Easy nLC 1200 chromatography system (Thermo Scientific, USA). For the DDA library generation, peptides were first loaded onto an EASY-Spray C18 Trap column (Thermo Scientific, USA,75 μm x 2 cm, 3 μm particle size), and subsequently separated on an EASY-Spray C18 LC Analytical Column (Thermo Scientific, USA, 75 μm x 25 cm, 2 μm particle size). A linear gradient of buffer B (80% acetonitrile, 0.1% formic acid) was applied at a flow rate of 250 nL/min over 90 min. The mass spectrometer was operated in positive ion mode, scanning in the range of 300–1800 m/z. The resolution for MS1 scans was set at 60,000 at 200 m/z, with an automatic gain control target of 3e6 and a maximum injection time of 25 ms. The top 20 most intense ions were fragmented using higher-energy collisional dissociation (HCD) with a normalized collision energy of 30 eV. MS2 scans were conducted at a resolution of 15,000, with an AGC target of 5e4 and a maximum IT of 25 ms.

For DIA analysis, each peptide sample was analyzed in DIA mode with one full MS-SIM scan and 30 DIA scans, covering a mass range of 350–1800 m/z. The full MS-SIM scan was conducted at a resolution of 120,000 (200 m/z), with an AGC target of 3e6 and a maximum IT of 50 ms. DIA scans were performed with a resolution of 15,000, an AGC target of 3e6, and a normalized collision energy of 30 eV. The runtime was 90 min, with a linear gradient of buffer B (80% acetonitrile and 0.1% formic acid) at a flow rate of 250 nL/min. Data analysis was performed using Spectronaut™ software, provided by Biognosys (Switzerland). QC samples were injected at the beginning and after every 6 runs to monitor the performance of the mass spectrometer. Detailed procedures for sample preparation and Mass Spectrometry Assay have been previously published [17].

### Clinical data collection

Demographic information, such as age and body mass index (BMI), was recorded. Blood samples were collected to measure fasting plasma glucose (FPG), fasting blood glucose (FBG), fasting insulin (FINS), cholesterol (CHO), triglycerides (TG), high-density lipoprotein (HDL), and low-density lipoprotein (LDL). The homeostasis model assessment of insulin resistance (HOMA-IR) was calculated using the formula: FPG (mmol/L) x FINS (µIU/mL)/22.5, with values exceeding 2.6 indicating insulin resistance. Reproductive outcomes include live birth and pregnancy loss. Live birth was defined as the birth of a living child after 24 weeks of gestation. Pregnancy loss included both biochemical pregnancy loss and clinical miscarriage. Patients in this study conceived by ART. Gestational time was estimated in weeks.

### Identification of DEPs

In the analysis of the initial dataset of proteins, we performed data normalization using the ‘normalizeBetweenArrays’ function to ensure uniformity across samples. Principal Component Analysis (PCA) was applied to evaluate variance within the dataset and distinguish between control and PCOS groups. To visualize DEPs differential expression, the ‘ggplot2’ package was used to create heat maps and volcano maps using the ‘limma’ package [18]. It was determined in this study that DEPs were statistically significant with the adjusted *p* value < 0.05 and |log2FC|>0.585.

### Functional enrichment analysis

Using the ‘clusterProfiler’ program [19], DEPs were subjected to Gene Ontology (GO) enrichment analysis and Kyoto Encyclopedia of Genes and Genomes (KEGG) pathway enrichment analysis. Adjust *p*-value < 0.05 were considered significantly enriched. In addition, the bar graph also shows the GO items as well as the KEGG pathways.

### Identification and analysis of prognostic metabolism-related proteins

Metabolism protein-coding genes sets were collected from human MSigDB collections(https://www.gsea-msigdb.org/). 947 metabolism-related protein-coding genes (shown in supplementary Table 1) were identified and intersected with 4425 expression proteins to obtain candidate prognostic proteins. Univariate Cox regression analyses with *p*-values below a predetermined threshold (*p* < 0.01) were considered potentially significant and selected for further analysis. LASSO regression, performed using the “glmnet” package in R [20] with ten-fold cross-validation, was then applied for further refinement and to construct the final model. Specifically, the λ value that minimized the cross-validation error was selected, ensuring that more candidate proteins were retained for an accurate prognostic model. Finally, to establish and visualize a protein-protein interaction (PPI) network for the metabolism differential proteins, we utilized the STRING database (available at https://cn.string-db.org/). For the correlation analysis of the DEPs, we employed the R packages ‘corrplot’, ‘reshape2’, and ‘igraph’ to effectively visualize the relationships based on their expression levels.

### Model construction and evaluation

Based on the above identified metabolism-related proteins, the metabolism signature formula was used as follows: Metabolism signature(PCOS) = ceof (Metabolism protein)∗expr(Metabolism protein). Metabolism signature (PCOS) represents a prognostic risk score, ceof (Metabolism protein) represents prognostic metabolism-related protein’s risk coefficient. And expr (Metabolism protein) is the expression of the screened metabolism-related proteins. Using the R package ‘survival,’ PCOS patients were classified into high-risk and low-risk groups based on their median risk score. The heatmap was generated to visualize the expression patterns of the identified metabolism-related proteins across high-risk and low-risk groups of PCOS patients. The boxplots were used to compare the expression levels of the ten most significant proteins between high-risk and low-risk groups using the t-test. The Kaplan-Meier method was then utilized to estimate reproductive outcomes. Additionally, the R package ‘survivalROC’ and time-dependent receiver operating characteristic (timeROC) curves were used to assess the reproductive outcomes of the Metabolism signature risk model. Decision curve analysis (DCA) was performed to evaluate the net benefits of the Metabolism signature risk model in comparison to various clinical predictors (including age, BMI, and IR) using the R package ‘ggDCA’. Calibration curves were also employed to assess the model’s performance.

### Correlation analysis

To investigate the correlation between clinical data and prognostic proteins, we performed correlation analysis using the R package ‘Hmisc’. The clinical data included variables such as BMI, age, and serum lipid levels. Pearson’s correlation coefficient was calculated to evaluate the relationships between these clinical variables and the expression levels of the identified prognostic proteins. Significant correlations (*p* < 0.05) were further visualized using scatter plots to illustrate the nature of these relationships.

### Statistical analysis

Statistical analyses were conducted using R software (version 4.4.0, https://www.R-project.org). Clinical data were expressed as mean ± standard or median (interquartile range), based on their distribution. Group differences were evaluated using suitable statistical tests, with *p* < 0.05 considered significant.

## Results

**Fig. 1:**
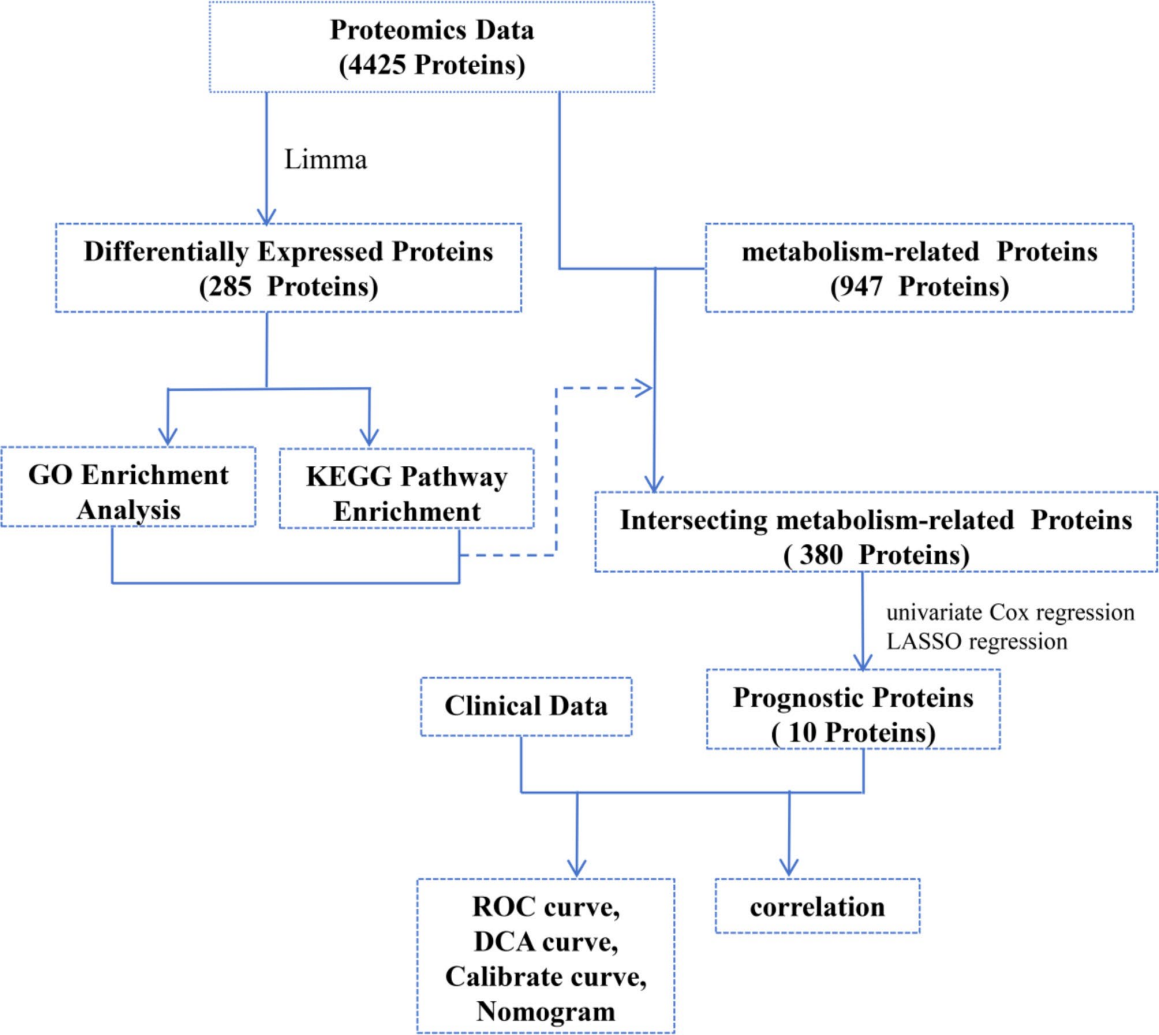
illustrates the workflow of proteomics data analysis

Figure 1 provides a clear step-by-step analysis, outlining the transition from initial data exploration to model construction. In this study, we started with an initial dataset of 4425 proteins and identified 285 DEPs. Simultaneously, we identified 947 metabolism-related proteins and intersected these with the 4425 proteins, resulting in 380 proteins. To identify key proteins linked to the prognosis of polycystic ovary syndrome (PCOS), we conducted univariate Cox regression analysis followed by LASSO regression, which ultimately identified 10 prognostic proteins. These prognostic proteins, combined with clinical data, were used to develop predictive models. The performance of the models was evaluated using ROC curves, DCA, calibration curves, and nomograms.

### Participant clinical characteristics

Table 1 provides an overview of the clinical characteristics of PCOS patients and control subjects. The PCOS group exhibited significantly higher BMI, FINS, and HOMA-IR compared to the control group, indicating greater metabolic disturbances in PCOS patients. The PCOS group also showed trends of higher cholesterol (CHO) and triglycerides (TG) levels, along with lower high-density lipoprotein (HDL) levels, although these differences were not statistically significant. Moreover, the rate of pregnancy loss was higher in the PCOS group compared to the control group, highlighting the increased reproductive challenges faced by women with PCOS. These reproductive issues may be linked to both metabolic disturbances and underlying endometrial dysfunction. Furthermore, the lower live birth rate observed in the PCOS group emphasizes the need for targeted interventions to improve reproductive outcomes in these patients.

**Table 1:** Participant clinical characteristics of patients with PCOS and controls

Variables	Total (*n* = 40)	Control (*n* = 7)	PCOS (*n* = 33)	*p*
Age (year)	26.0 ± 3.1	27.0 ± 2.9	25.8 ± 3.1	0.365
BMI (kg/m^2^)	23.7 ± 3.8	21.4 ± 2.8	24.2 ± 3.8	0.048
FPG (mmol/L)	5.1 ± 0.6	4.5 ± 0.4	5.2 ± 0.5	0.002
FINS (mIU/mL)	13.9 (9.5, 25)	7.3 (6.9, 11.6)	16.2 (9.8, 25.8)	0.016
HOMA-IR	3.1 (2.1, 5.5)	1.7 (1.3, 2.2)	3.9 (2.4, 6.3)	0.004
CHO (mmol/L)	4.0 ± 0.7	3.6 ± 0.7	4.1 ± 0.7	0.212
TG (mmol/L)	1.1 (0.8, 1.9)	0.9 (0.8, 0.9)	1.3 (0.9, 2)	0.109
HDL (mmol/L)	1.3 (1.1, 1.5)	1.4 (1.2, 1.5)	1.3 (1.1, 1.4)	0.285
LDL (mmol/L)	2.6 ± 0.7	2.2 ± 0.5	2.7 ± 0.7	0.056
Pregnancy loss, *n* (%)				0.043
Live birth (%)	27 (67.5)	7 (100)	20 (60.6)	
Pregnancy loss (%)	13 (32.5)	0 (0)	13 (39.4)	

BMI: Body mass index, FPG: Fasting plasma glucose, FINS: Fasting insulin, HOMA-IR: Homeostasis model assessment of insulin resistance, CHO: cholesterol, TG: triglyceride, HDL: high-density lipoprotein, LDL: low-density lipoprotein. *p* < 0.05 was considered statistically significant

### Proteomic data analysis and screen differential expression proteins

**Fig. 2:**
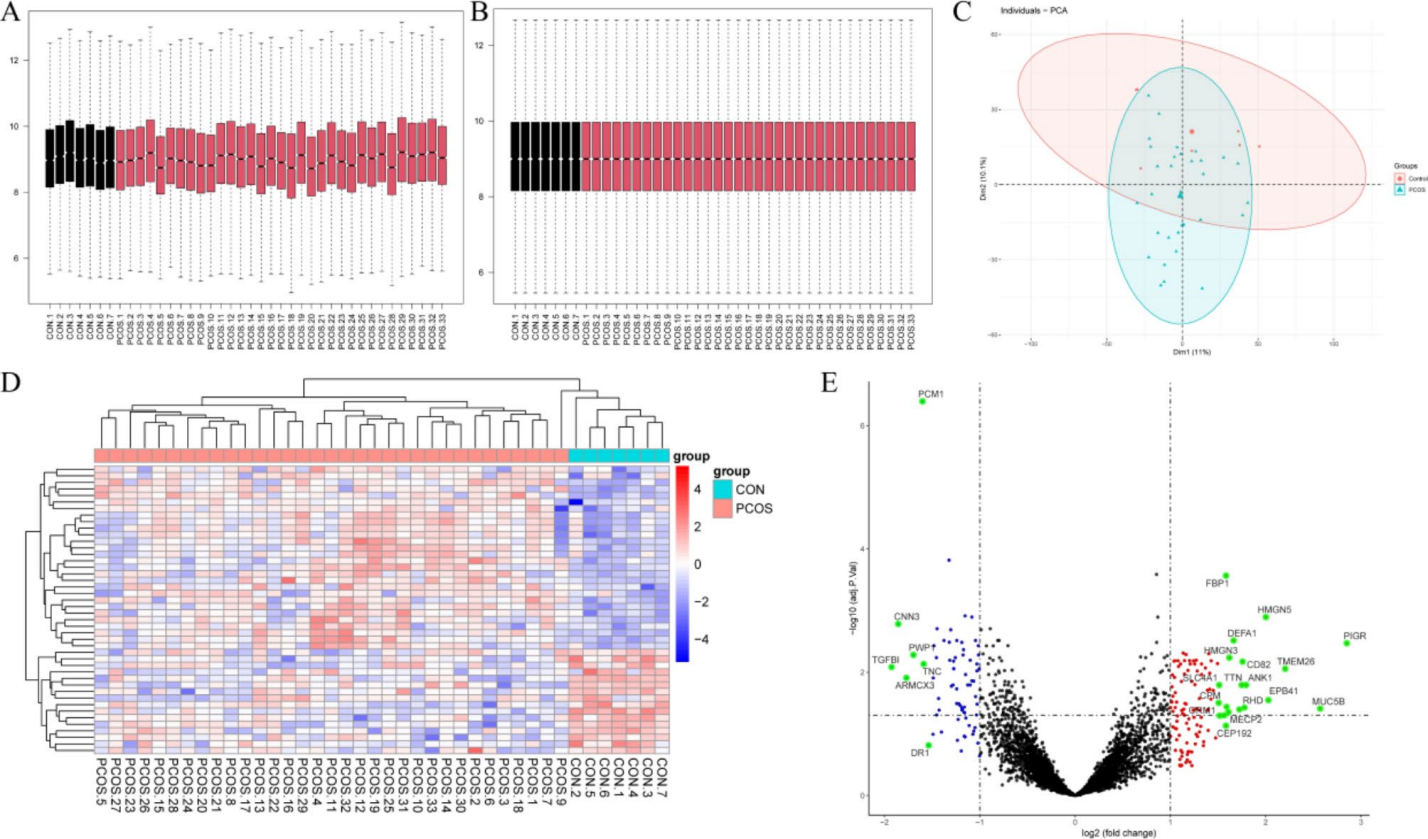
Differential Expression Analysis of Proteomic Data. (**A**) Boxplot of proteomic data before normalization. The black boxes represent the control group, and the red boxes represent the PCOS group. (**B**) Boxplot of proteomic data after normalization, which showing more uniform distribution. (**C**) Principal Component Analysis (PCA) plot of the proteomic data, demonstrating the separation between the control group (blue) and the PCOS group (red). (**D**) Heatmap of DEPs between the control and PCOS groups. The red and blue colors indicate higher and lower expression levels, respectively. (**E**) Volcano plot of DEPs, with the x-axis representing the log2 fold change and the y-axis representing the -log10 *p*-value. Red and blue dots represent significantly upregulated and downregulated proteins, respectively, while green dots highlight the most significantly different proteins

From the initial dataset of 4425 proteins, we examined the raw data distribution and applied normalization to ensure uniformity across samples (Fig. 2A-B). The boxplots clearly show that normalization reduces variability and aligns the data distribution more closely between the control and PCOS groups. Principal Component Analysis (PCA) was conducted to illustrate the overall variance in the data and to distinguish between the control and PCOS groups. As shown in Fig. 2C, the PCA plot illustrates a distinct separation between the two groups. Finally, 285 differentially DEPs were identified between the PCOS and control groups, as illustrated by the heatmap in Fig. 2D and the volcano plot in Fig. 2E. The heatmap shows clustering of the DEPs, with distinct expression patterns between the two groups, supporting the findings from PCA that highlight the biological differences between PCOS and control samples. The volcano plot further visualizes the significant upregulated and downregulated proteins, which are critical to understanding the underlying metabolic processes in PCOS.

### Differentially expressed proteins functional enrichment analysis

**Fig. 3:**
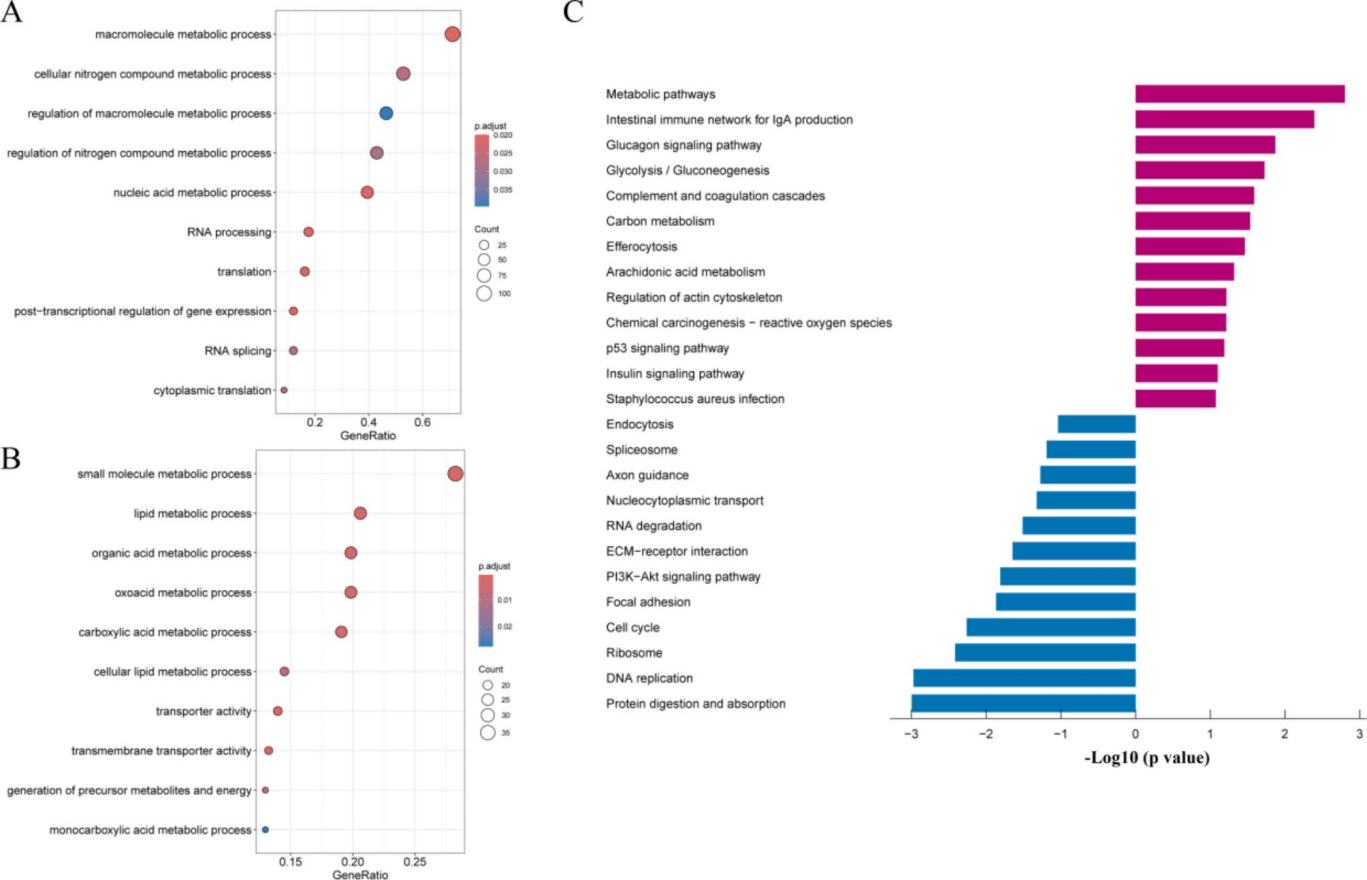
GO and KEGG Enrichment Analysis of Differentially Expressed Proteins. (**A**) Gene Ontology (GO) enrichment analysis of downregulated proteins. The size of the bubbles represents the count of proteins, and the color gradient indicates the adjusted *p*-value. (**B**) GO enrichment analysis of upregulated proteins. (**C**) KEGG pathway enrichment analysis of differentially expressed proteins. The bar plot displays significantly enriched pathways, with blue bars representing downregulated proteins and red bars representing upregulated proteins. The x-axis representing the -log10 (*p*-value)

To further explore the biological significance of the DEPs in PCOS, we performed GO and KEGG pathway enrichment analyses (Supplementary Table e2). GO enrichment analysis of downregulated proteins (Fig. 3A) indicated significant involvement in macromolecule metabolic processes, cellular nitrogen compound metabolic processes, and the regulation of metabolic processes. These findings suggest a suppression of essential metabolic functions in PCOS, potentially contributing to impaired cellular homeostasis and energy production. On the other hand, GO analysis of upregulated proteins (Fig. 3B) indicated significant enrichment in small molecule metabolic processes, lipid metabolic processes, and transporter activity. This suggests that certain metabolic pathways, particularly those related to lipid and small molecule metabolism, are activated in PCOS, possibly contributing to the dysregulated lipid profile commonly observed in these patients. The KEGG pathway enrichment analysis (Fig. 3C) provided further insights into the functional pathways involved. Upregulated proteins were significantly enriched in pathways such as metabolic pathways, glucagon signaling, and glycolysis/gluconeogenesis, indicating a shift towards increased energy mobilization and metabolic dysfunction. Downregulated proteins were involved in pathways such as spliceosome, axon guidance, nucleocytoplasmic transport, and RNA degradation. The downregulation of these pathways suggests a disruption in cellular communication and RNA processing in PCOS, which may further exacerbate reproductive and metabolic disturbances.

### Identification and analysis of prognostic metabolism-related proteins

**Fig. 4:**
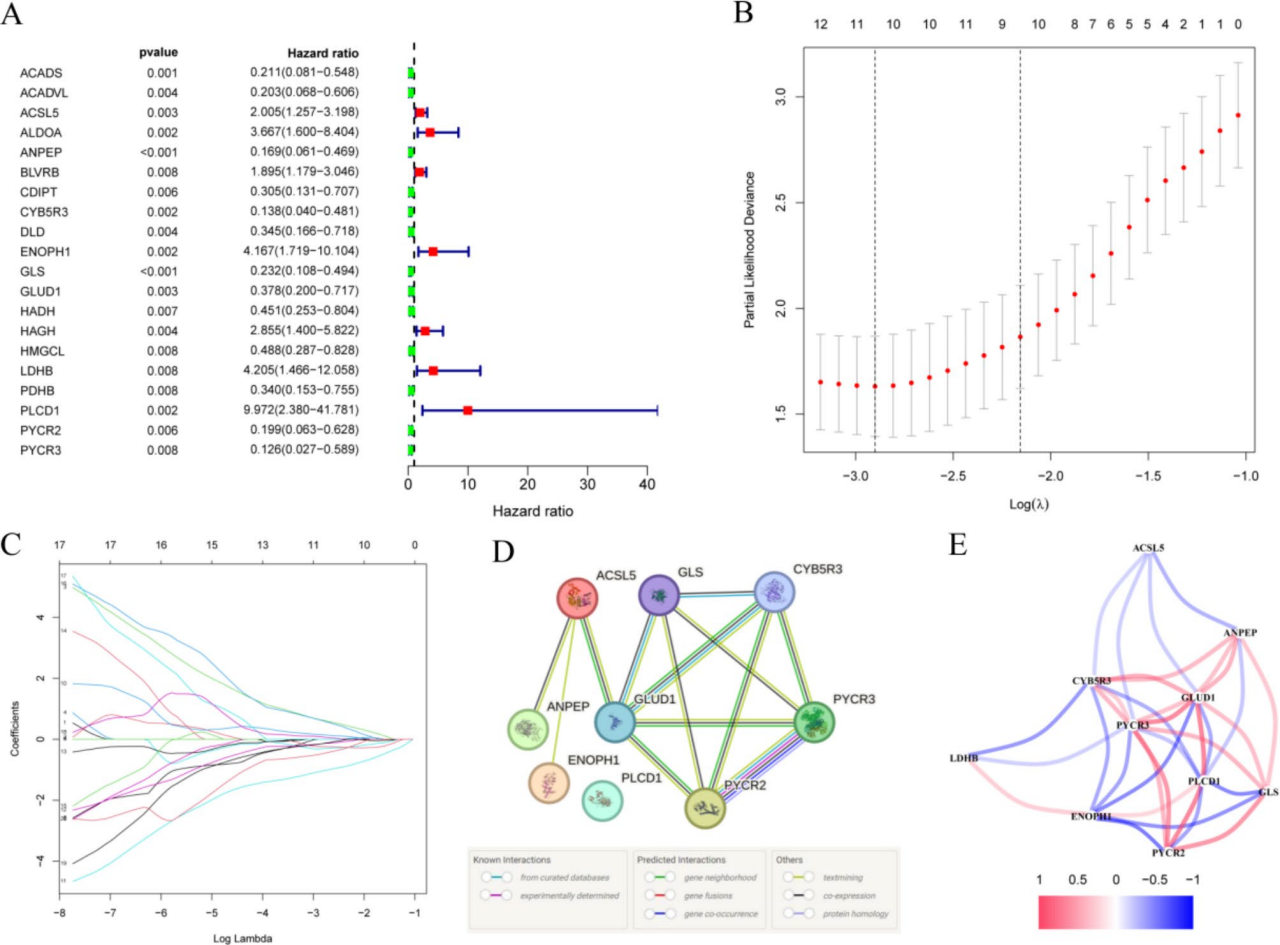
Identification and Analysis of Prognostic Metabolism-Related Proteins. (**A**) Forest plot showing the Univariate Cox regression analysis of metabolism-related proteins. The x-axis represents the hazard ratio, and the y-axis lists the proteins with significant associations. The x-axis represents the hazard ratio, while the y-axis lists the proteins that show significant associations with the outcome. (**B**) Partial likelihood deviance plot from the LASSO regression model, displaying the tuning parameter (lambda) selection process. (**C**) LASSO coefficient profiles of the metabolism-related proteins. (**D**) The protein-protein interaction network of these potential Metabolism-related Proteins. (**E**) The expressions of screened Metabolism DEPs were used to establish a correlated network, with blue lines indicating negative correlations and red lines indicating positive correlations. The thickness of the lines represents the strength of the correlations

Univariate Cox regression analysis, followed by LASSO regression (Fig. 4A-C), identified 20 candidate metabolism-related proteins significantly associated with reproductive outcomes, which were further refined to 10 key prognostic proteins: ACSL5, ANPEP, CYB5R3, ENOPH1, GLS, GLUD1, LDHB, PLCD1, PYCR2, and PYCR3. These proteins were considered to have the most substantial association with reproductive outcomes and were selected for further analysis. To explore the functional relationships among these 10 key prognostic proteins, a protein-protein interaction (PPI) network was constructed using the STRING database (Fig. 4D). The network reveals multiple interactions among the selected proteins, suggesting their involvement in interconnected metabolic pathways. Figure 4E shows the correlation network of the identified metabolism-related DEPs. This network visualizes the correlations based on expression levels. Strong positive correlations may indicate cooperative roles in metabolic processes, whereas negative correlations suggest opposing regulatory effects.

### Construct the risk prognostic model

**Fig. 5:**
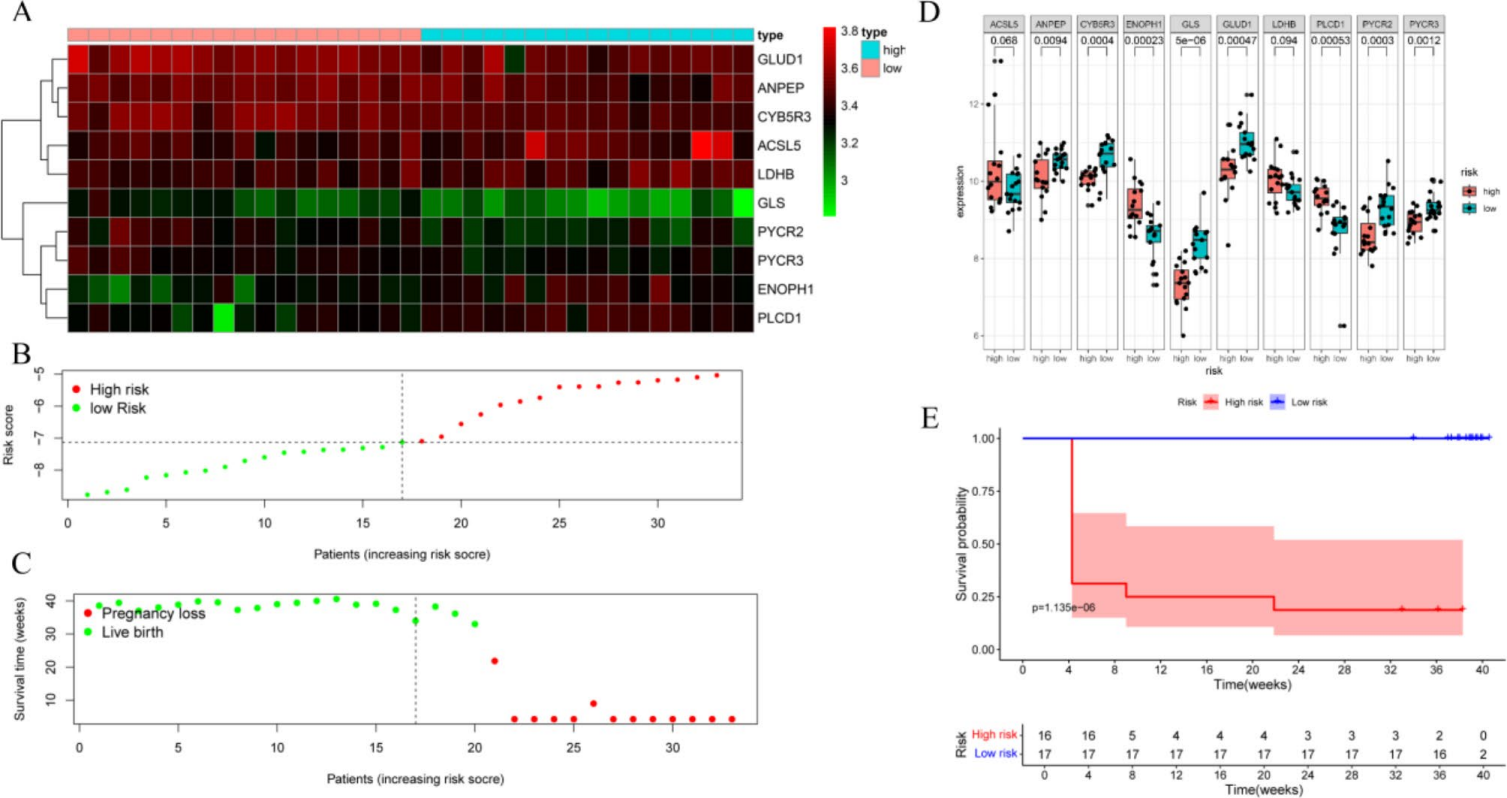
Construct the Risk Prognostic Model. (**A**) Heatmap showing the expression levels of selected metabolism-related proteins in high-risk and low-risk PCOS groups. Red indicates higher expression, and green indicates lower expression. (**B**) Risk score distribution plot, with high-risk patients represented by red dots and low-risk patients by green dots. (**C**) Survival status plot, showing the relationship between risk scores and pregnancy outcomes. Red dots represent pregnancy loss, and green dots represent live birth. (**D**) The boxplots of the expression levels of the ten significant proteins between high-risk and low-risk groups. (**E**) Kaplan-Meier survival curve comparing the high-risk and low-risk groups. The y-axis represents survival probability, and the x-axis shows time in weeks. The red line indicates the high-risk group, and the blue line indicates the low-risk group

We constructed the risk prognostic model using the ten metabolism-related proteins previously screened. As a result of prognostic data and ten metabolism-related proteins, the risk prognostic signature was developed as follows: Risk score = 0.52 ×expr (ACSL5) -0.28×expr (ANPEP) -0.25×expr (CYB5R3) + 0.11×expr (ENOPH1) -0.94×expr (GLS) -0.10×expr (GLUD1) + 0.47×expr (LDHB) + 0.03×expr (PLCD1)-0.06×expr (PYCR2)-0.36×expr (PYCR3). The heatmap illustrates the expression levels of the selected metabolism-related proteins in high-risk and low-risk PCOS groups. This visualization confirms distinct expression patterns between the two groups, with specific proteins being markedly upregulated or downregulated (Fig. 5A). This differential expression suggests that these metabolism-related proteins may play critical roles in modulating the risk of adverse reproductive outcomes in PCOS. The risk score distribution plot categorizes patients into high-risk and low-risk groups based on their risk scores (Fig. 5B). Figure 5C illustrated the survival status plot correlates risk scores with pregnancy outcomes, showing a clear distinction between live birth and pregnancy loss. The clear separation between the two groups emphasizes the effectiveness of the risk score in predicting reproductive success, highlighting the clinical utility of the prognostic model. Expression levels of the 10 selected proteins were compared between high-risk and low-risk PCOS groups. Boxplots showing the expression differences of 10 selected proteins between high-risk and low-risk PCOS group (Fig. 5D). The Kaplan-Meier survival curve demonstrates a significant difference in survival probability between high-risk and low-risk groups, with the high-risk group having a markedly lower survival probability (Fig. 5E). This indicates the strong prognostic value of the identified metabolism-related proteins in predicting adverse outcomes in PCOS.

### Evaluation of the risk prognostic model

**Fig. 6:**
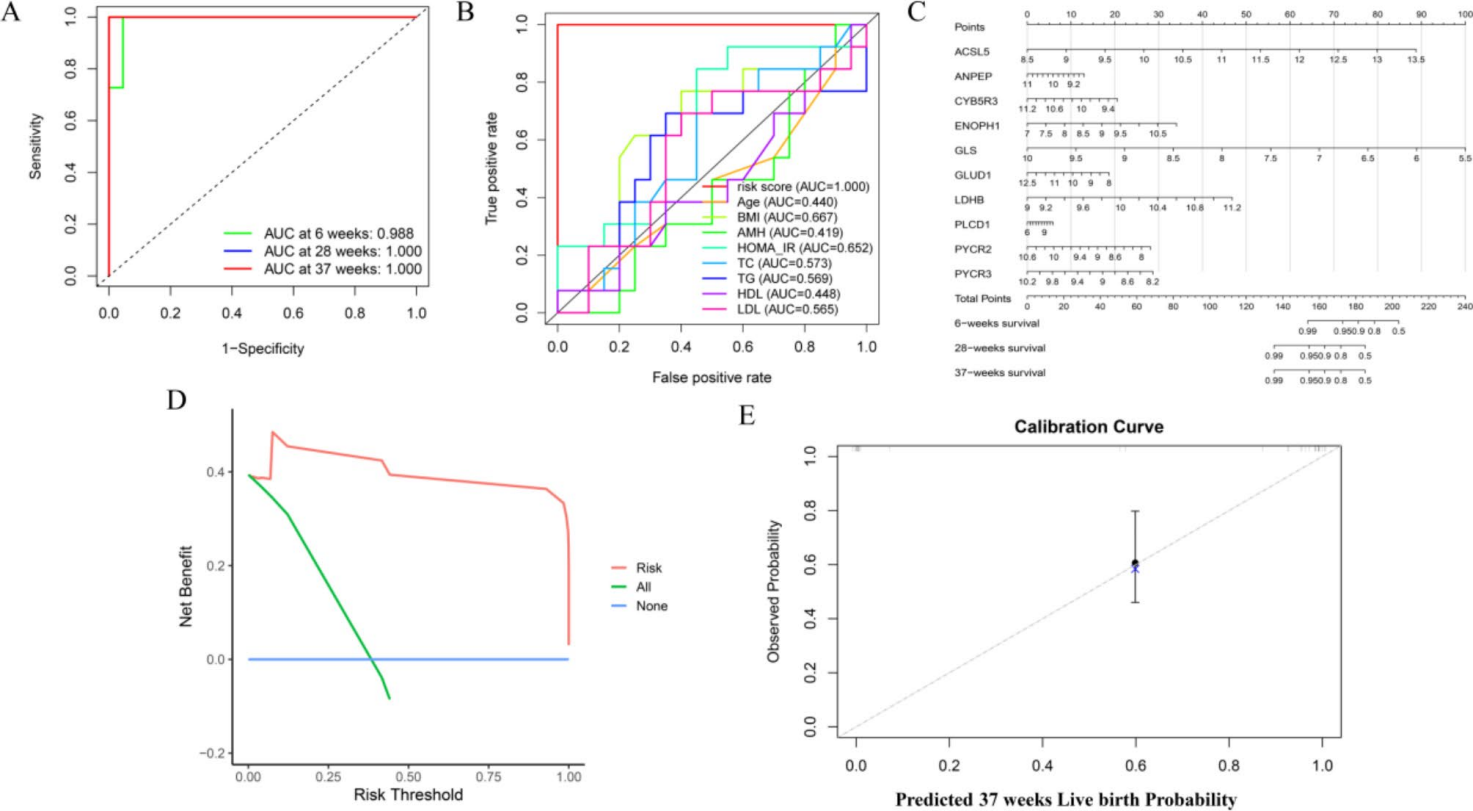
Prognostic Analysis of Selected Metabolism-Related Proteins. (**A**) Receiver operating characteristic (ROC) curves for predicting outcomes at 6 weeks, 28 weeks, and 37 weeks using the 10 selected proteins. The area under the curve (AUC) values are shown for each time point. (**B**) ROC curves comparing the predictive performance of clinical data and the selected proteins. (**C**) Nomogram for predicting the probability of live birth at 6 weeks, 28 weeks, and 37 weeks based on the 10 selected proteins. (**D**) Decision curve analysis (DCA) for predicting 37-week outcomes, showing the net benefit of the risk prediction model. (**E**) Calibration curve for predicting 37-week live birth probability, comparing predicted probabilities with observed outcomes

To evaluate the performance of our prognostic model, we assessed its ability to predict pregnancy outcomes at different time points: 6, 28, and 37 weeks. The ROC curves for these predictions demonstrated excellent performance, with AUC values of 0.988 at 6 weeks, and perfect scores of 1.000 at both 28 and 37 weeks (Fig. 6A). The model’s predictive performance was compared to traditional clinical variables, including age, BMI, AMH, HOMA-IR, and lipid profiles (Fig. 6B). The protein-based model significantly outperformed all individual clinical markers, with an AUC of 1.000 compared to AUC values ranging from 0.419 to 0.667 for the clinical features. This highlights the superiority of the protein-based model in accurately predicting live birth outcomes, demonstrating its potential as a more reliable tool for risk assessment in PCOS compared to conventional clinical indicators. A nomogram integrating the 10 proteins was developed to predict live birth probability at 6, 28, and 37 weeks (Fig. 6C). The nomogram offers a practical and individualized risk prediction tool that can be utilized in clinical settings, providing a user-friendly way to estimate the likelihood of successful pregnancy outcomes based on protein expression levels. Figure 6D presents the decision curve analysis (DCA) for 37 weeks live birth prediction, showing a clear net benefit for using the protein-based risk model across a range of threshold probabilities. The calibration curve (Fig. 6E) confirmed the accuracy of the model in predicting live birth probability at 37 weeks, with observed outcomes closely matching the predicted probabilities.

### Correlation analysis between clinical data and prognostic proteins

**Fig. 7:**
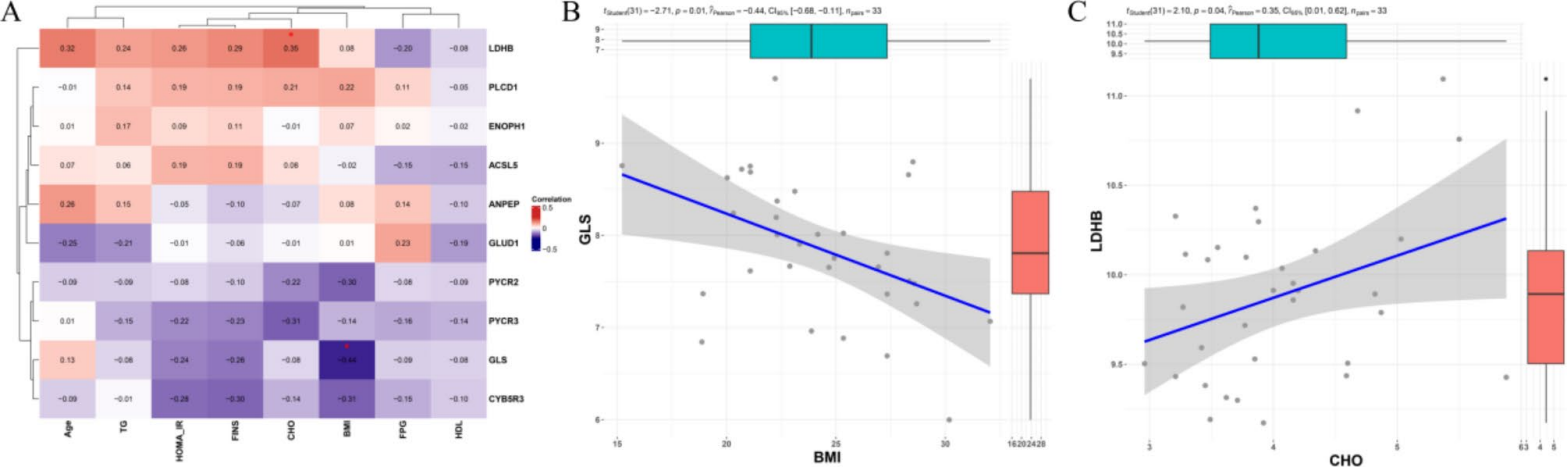
The correlation between clinical data and prognostic proteins. (**A**) Heatmap showing the correlation between clinical data and prognostic proteins identified from the study. Pearson’s correlation coefficients are presented, with significant correlations (*p* < 0.05) marked by *. (**B**) Scatter plot illustrating the significant negative correlation between BMI and GLS, with a fitted regression line (*r* = -0.44, *p* = 0.01). (C) Scatter plot showing the significant positive correlation between CHO and LDHB, with a fitted regression line (*r* = 0.35, *p* = 0.04)

The correlation analysis was visualized in a heatmap (Fig. 7A), highlighting the relationships between clinical variables (such as BMI, age, and serum lipid levels) and the expression levels of the prognostic proteins. This suggests that higher BMI is associated with reduced GLS expression, potentially indicating metabolic dysregulation linked to impaired glutamine metabolism in individuals with higher body weight. BMI exhibited a significant negative correlation with the expression of GLS (*r* = -0.44, *p* = 0.01), as depicted in the scatter plot (Fig. 7B). Additionally, CHO showed a significant positive correlation with the expression of LDHB (*r* = 0.35, *p* = 0.04), as shown in Fig. 7C. Elevated LDHB levels in individuals with higher cholesterol could reflect an increased reliance on anaerobic glycolysis, which might be associated with metabolic stress or lipid dysregulation in PCOS patients.

## Discussion

Polycystic ovary syndrome (PCOS) is a complex endocrine disorder characterized by a range of reproductive, metabolic, and psychological symptoms [21]. Beyond anovulation, which is commonly associated with infertility in PCOS patients, endometrial defects leading to recurrent miscarriages and implantation failures are also significant contributors to reproductive challenges [22, 23]. Improving the receptivity of the endometrium in PCOS patients can reduce adverse reproductive outcomes, such as recurrent miscarriages and implantation failures, particularly in those who have regained ovulatory function [24, 25]. This study aimed to uncover the significance of metabolism-related proteins in the prognosis of PCOS through comprehensive endometrium proteomic analysis and clinical data integration. Our findings provide novel insights into the metabolic mechanisms underlying PCOS and highlight potential biomarkers for prognosis and personalized treatment.

Endometrial receptivity and reproductive outcomes in PCOS patients are likely influenced by alterations in multiple signaling pathways during disease progression [26–28]. The identification of DEPs between PCOS patients and control subjects underscores the critical role of metabolic dysfunction in the pathogenesis of PCOS. GO and KEGG enrichment analyses highlighted the involvement of these DEPs in lipid and glucose metabolism, which are known to be dysregulated in PCOS [29, 30]. Previous studies have consistently highlighted the association between PCOS and metabolic dysfunction [31, 32]. For instance, Ying Yu et al. used DIA proteomics to identify significant changes in serum proteins between PCOS patients and healthy controls. Their study identified 285 DEPs, with downregulated proteins enriched in processes such as cell adhesion, coagulation, and inflammatory responses, and upregulated proteins involved in antioxidant activity and cellular detoxification [33]. Similarly, Jun Li et al. conducted a comprehensive analysis to identify proteomic alteration of endometrial tissues in PCOS. Their research emphasized the role of metabolic and immune dysregulation in the pathophysiology of PCOS, identifying proteins involved in metabolism, inflammation, and cell adhesion molecules [34]. Those finding reveals the intricate link between metabolic pathways and endometrial function in PCOS, suggesting that targeting metabolic dysfunction could be a potential strategy to improve reproductive outcomes in PCOS patients.

Accurately predicting reproductive outcomes in PCOS patients using traditional clinical features remains challenging, and there is a notable scarcity of specific biomarkers for PCOS [35]. Therefore, there is an urgent need for more precise prognostic models for PCOS patients. Univariate Cox regression analysis, followed by LASSO regression identified 10 key metabolism-related proteins significantly associated with reproductive outcomes in PCOS: ACSL5, ANPEP, CYB5R3, ENOPH1, GLS, GLUD1, LDHB, PLCD1, PYCR2, and PYCR3. Previous studies have demonstrated that these 10 proteins are closely related to metabolism diseases, but their roles in PCOS are less well-studied. ACSL5 plays a crucial role in fatty acid metabolism by catalyzing the formation of acyl-CoA from fatty acids. Elevated ACSL5 levels in PCOS patients suggest an alteration in lipid metabolism, which is consistent with the known metabolic disturbances in PCOS, such as increased lipogenesis and altered fatty acid oxidation [36]. ANPEP, also known as CD13, is involved in protein processing and amino acid metabolism [37]. It has been implicated in various physiological processes, including angiogenesis and immune responses [38, 39]. The significant association of ANPEP with PCOS prognosis may indicate its role in modulating inflammatory and metabolic pathways, contributing to the disease’s pathophysiology. CYB5R3 is essential for electron transport and lipid metabolism [40]. Its differential expression in PCOS patients highlights the importance of redox balance and mitochondrial function in the disorder. Disruptions in these processes can lead to oxidative stress, a known factor in PCOS pathogenesis. ENOPH1 is involved in the methionine salvage pathway and cellular stress responses [41]. Altered ENOPH1 levels in PCOS suggest a potential disruption in amino acid metabolism and cellular homeostasis, which could impact endometrial function and reproductive outcomes. GLS catalyzes the conversion of glutamine to glutamate, a key step in nitrogen metabolism. Elevated GLS levels in PCOS patients point to an increased demand for glutamate [42], which may be linked to altered energy metabolism and the insulin resistance commonly observed in PCOS. GLUD1 is critical for the oxidative deamination of glutamate, playing a role in both energy production and ammonia detoxification [43, 44]. Its association with PCOS prognosis suggests that disruptions in glutamate metabolism and mitochondrial function may contribute to the metabolic and reproductive abnormalities in PCOS. LDHB is involved in the conversion of pyruvate to lactate and vice versa, which is central to glucose metabolism and the Cori cycle [45]. The differential expression of LDHB in PCOS patients underscores the metabolic flexibility required to manage glucose and lactate levels, reflecting the broader metabolic dysregulation in PCOS. PLCD1 plays a role in phosphoinositide metabolism and intracellular signaling [46]. Its involvement in PCOS may be related to altered lipid signaling pathways, which are crucial for various cellular functions, including insulin signaling and energy homeostasis. PYCR enzymes are involved in proline biosynthesis, which are important for protein synthesis, redox balance, and cell cycle progression [47, 48]. The significant association of PYCR2 and PYCR3 with PCOS highlights the potential role of altered proline metabolism in the disorder, possibly affecting tissue remodeling and oxidative stress management.

The 33 PCOS patients were finally divided into high-risk and low-risk groups based on risk scores, revealing a significant difference in live birth rates between the two groups. We further demonstrated that a high-risk score is associated with adverse pregnancy outcomes. The time-dependent ROC curves for the model based on the 10 metabolism-related proteins effectively predicted pregnancy outcomes in PCOS patients. Although clinical indicators are more convenient to use, the prognostic model based on these 10 metabolism-related proteins showed significant clinical predictive efficacy compared to other clinical variables, including age, BMI, IR, and serum lipids. Decision Curve Analysis (DCA) indicated that applying this model provides a net benefit, highlighting its potential utility in clinical practice.

The correlation analysis between clinical data and the expression levels of prognostic proteins provides valuable insights into the potential mechanisms underlying the metabolic disturbances observed in PCOS. BMI was found to have a significant negative correlation with the expression of GLS (*r* = -0.44, *p* = 0.01). This suggests that higher BMI is associated with lower levels of GLS expression. GLS (Glutaminase) is an enzyme involved in glutamine metabolism, which plays a crucial role in cellular energy production and nitrogen metabolism [42]. Reduced GLS expression in individuals with higher BMI could indicate metabolic dysregulation, potentially affecting cellular energy homeostasis and contributing to the pathophysiology of PCOS. However, limited research on the relationship between GLS and BMI which deserves further study. Serum cholesterol levels (CHO) showed a significant positive correlation with the expression of LDHB (*r* = 0.35, *p* = 0.04). LDHB (Lactate Dehydrogenase B) is an enzyme involved in the glycolytic pathway, catalyzing the conversion of lactate to pyruvate [49]. The positive correlation indicates that higher cholesterol levels are associated with increased LDHB expression. Elevated LDHB levels may reflect enhanced glycolytic activity, potentially linked to insulin resistance [45]. The identified correlations between clinical variables and prognostic proteins underscore the complex interplay between metabolic and reproductive factors in PCOS. Metabolic dysfunctions, such as obesity and dyslipidemia, appear to influence the expression of key metabolic enzymes, which in turn may affect cellular functions and contribute to the clinical manifestations of PCOS. Understanding these relationships is crucial for developing targeted therapeutic strategies aimed at addressing the metabolic aspects of PCOS, ultimately improving patient outcomes.

The identified proteins provide valuable biomarkers for predicting adverse reproductive outcomes in PCOS. The integration of these proteins into a prognostic model demonstrated superior predictive performance compared to traditional clinical markers, with AUC values of 1.000 at both 28 and 37 weeks. The developed nomogram and decision curve analysis (DCA) further validate the clinical utility of this model, enabling personalized risk assessment and targeted interventions.

Despite the promising findings, this study has several limitations. The relatively small sample size, particularly of the control group, may limit the generalizability of the results. Additionally, we have not yet conducted foundational research to experimentally validate the identified biomarkers. Furthermore, our current DIA analysis uses a DDA-generated spectral library. In future studies, we can explore library-free methods, such as DiaNN and Spectronaut, to potentially enhance peptide detection. Moreover, exploring the interactions between these proteins and other metabolic pathways could provide a comprehensive understanding of the disease and inform the development of more effective treatments.

## Conclusion

This study identified 10 key metabolism-related proteins that are significantly associated with the prognosis of PCOS. The protein-based prognostic model offers a robust tool for predicting adverse reproductive outcomes for PCOS patients. Our findings underscore the importance of addressing metabolic dysfunction in PCOS and pave the way for future research into targeted interventions to improve reproductive and metabolic health outcomes in affected women.

## Electronic supplementary material

Below is the link to the electronic supplementary material.

Supplementary Material 1

Supplementary Material 2

## Data Availability

The datasets presented in this study can be found in online repositories. The names of the repository/repositories and accession numbers can be found below: The mass spectrometry proteomics data have been deposited to the ProteomeXchange consortium (http://proteomecentral.proteomexchange.org) via the iProX partner repository with the dataset identifier PXD032383.
